# Transformation to small-cell carcinoma as an acquired resistance mechanism to AZD9291: A case report

**DOI:** 10.18632/oncotarget.14506

**Published:** 2017-01-04

**Authors:** Lin Li, Hui Wang, Chao Li, Zheng Wang, Ping Zhang, Xu Yan

**Affiliations:** ^1^ Department of Oncology, Beijing Hospital, National Center of Gerontology, Beijing, China; ^2^ Department of Pathology, Beijing Hospital, National Center of Gerontology, Beijing, China

**Keywords:** NSCLC, EGFR-TKI, AZD9291, acquired resistance, transformation

## Abstract

AZD9291, a third-generation epidermal growth factor receptor (EGFR) tyrosine kinase inhibitor (TKI), benefits patients with T790M mutant non-small-cell lung cancer who fail treatment with first-generation EGFR TKIs. Acquisition of resistance to AZD9291 occurs inevitable and mechanisms need to be explored. We reported an advanced lung adenocarcinoma female with EGFR exon19 deletion treated on AZD9291 after failure of erlotinib and chemotherapy. Disease progressed again after 6 months treatment of AZD9291 with hepatic metastasis. Re-biopsy of the hepatic lesion showed histopathology transformation to small cell lung cancer, which harbored EGFR exon19 deletion. Therefore, small cell carcinoma transformation is one of potential resistance mechanisms to AZD9291 and regimen for small cell carcinoma may be one of the treatment options.

## INTRODUCTION

Epidermal growth factor receptor (EGFR) tyrosine kinase inhibitors (TKIs) play very important roles in treatment of advanced EGFR mutated non-small cell lung cancer (NSCLC). However, acquired resistance may develop inevitably, and T790M mutation accounts for approximately 60% of the resistance cases in first-generation TKIs treatment [1, 2]. Third-generation TKIs such as AZD9291 were effective against T790M mutated NSCLC, with overall response rate (ORR) of about 60%, but acquired resistance occur in about 10 months [3]. The mechanisms of acquired resistance to third-generation TKIs need to be explored. Here we reported a case of small cell lung cancer (SCLC) transformation post AZD9291 treatment as a resistance mechanism.

## CASE REPORT

**Figure 1:**
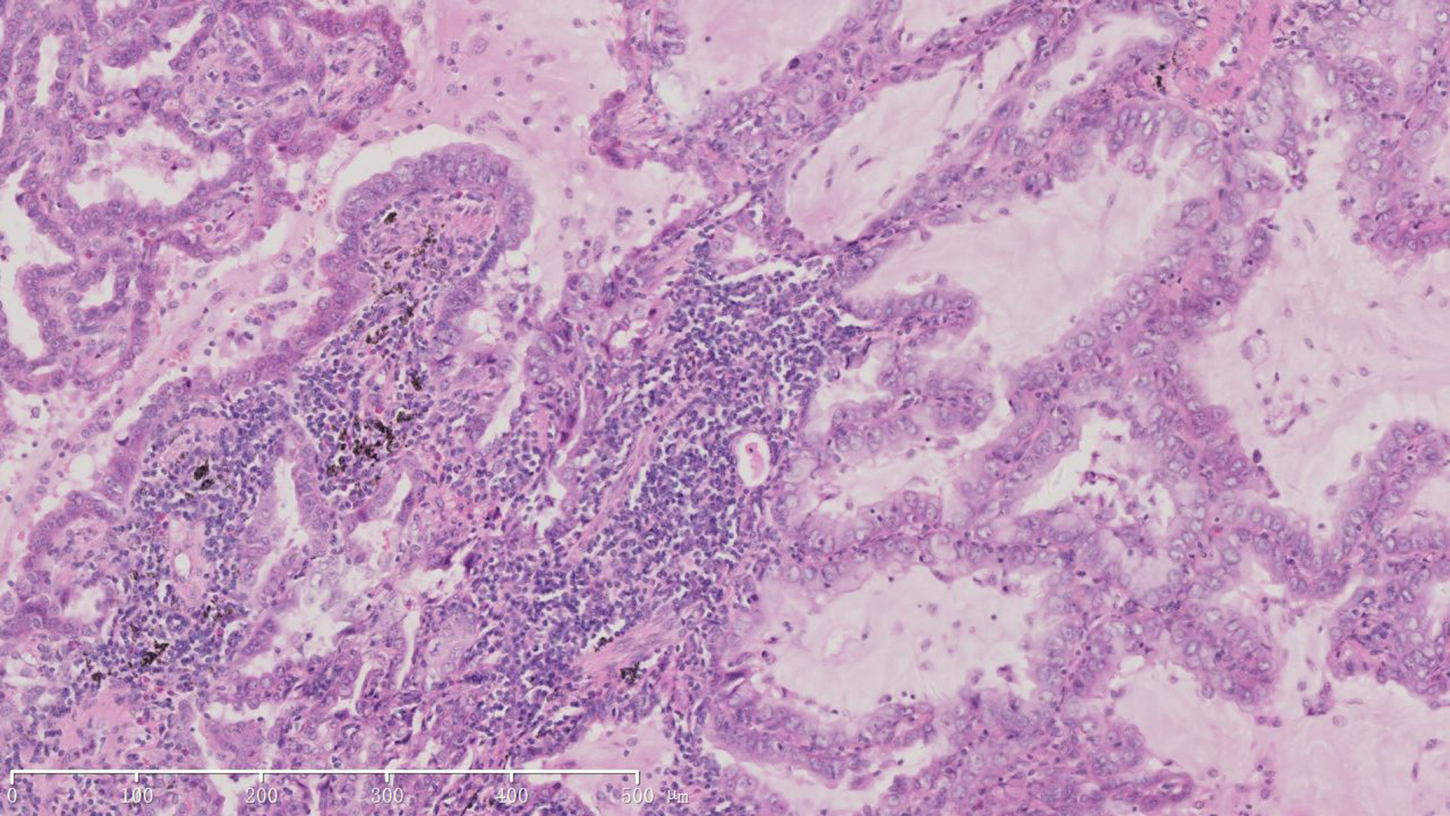
HE staining of surgical sample of lung showed histopathology of adenocarcinoma (X100)

**Figure 2:**
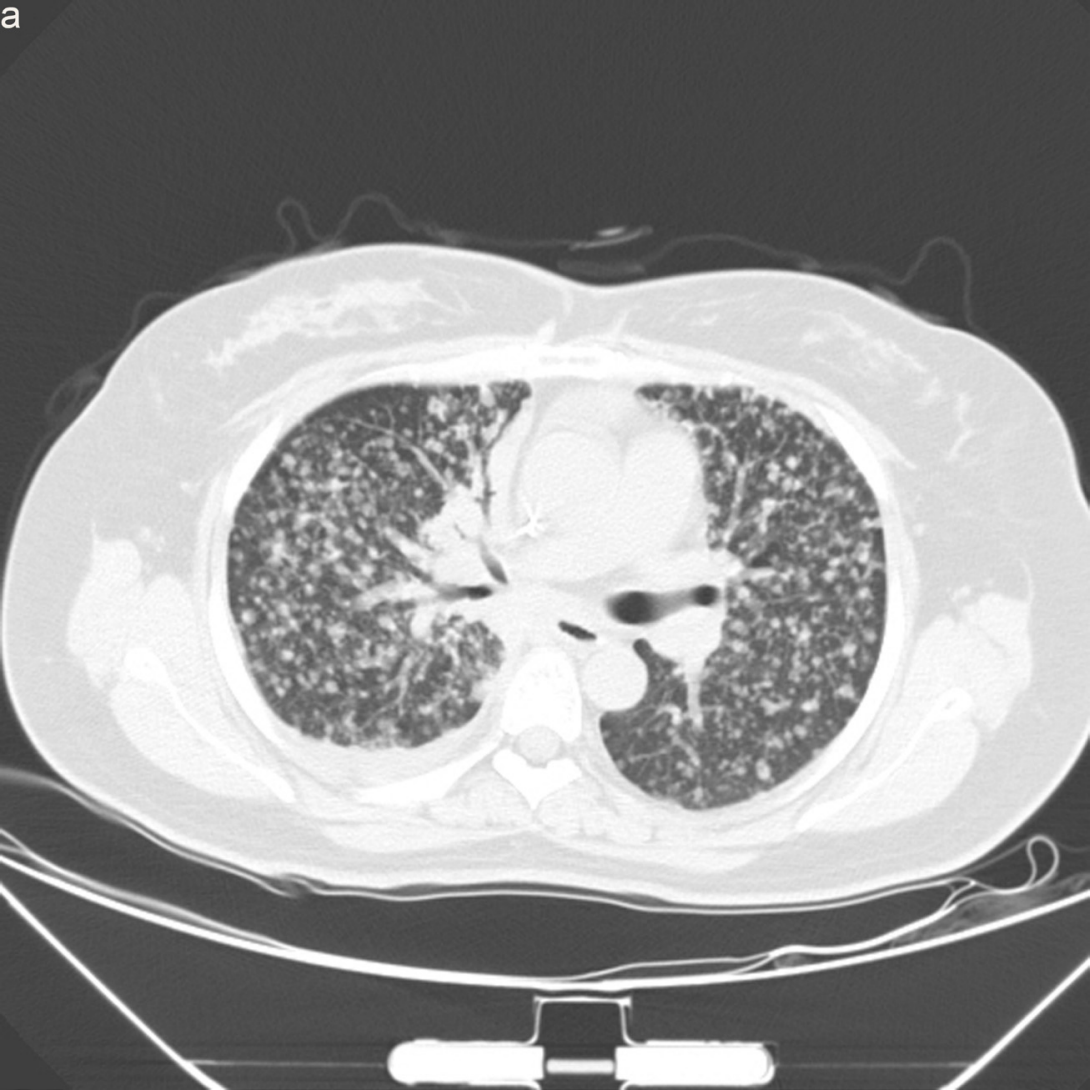
Computed tomography scan images of lung prior and post-AZD9291 treatment **a**. Disease progressed in Dec,2015 after treatment of erlotinib and chemotherapy, prior-AZD9291. Patient had symptoms of cough and short of breath. **b**. Partial response after one month of AZD9291 treatment. Symptoms were much relieved.

**Figure 3:**
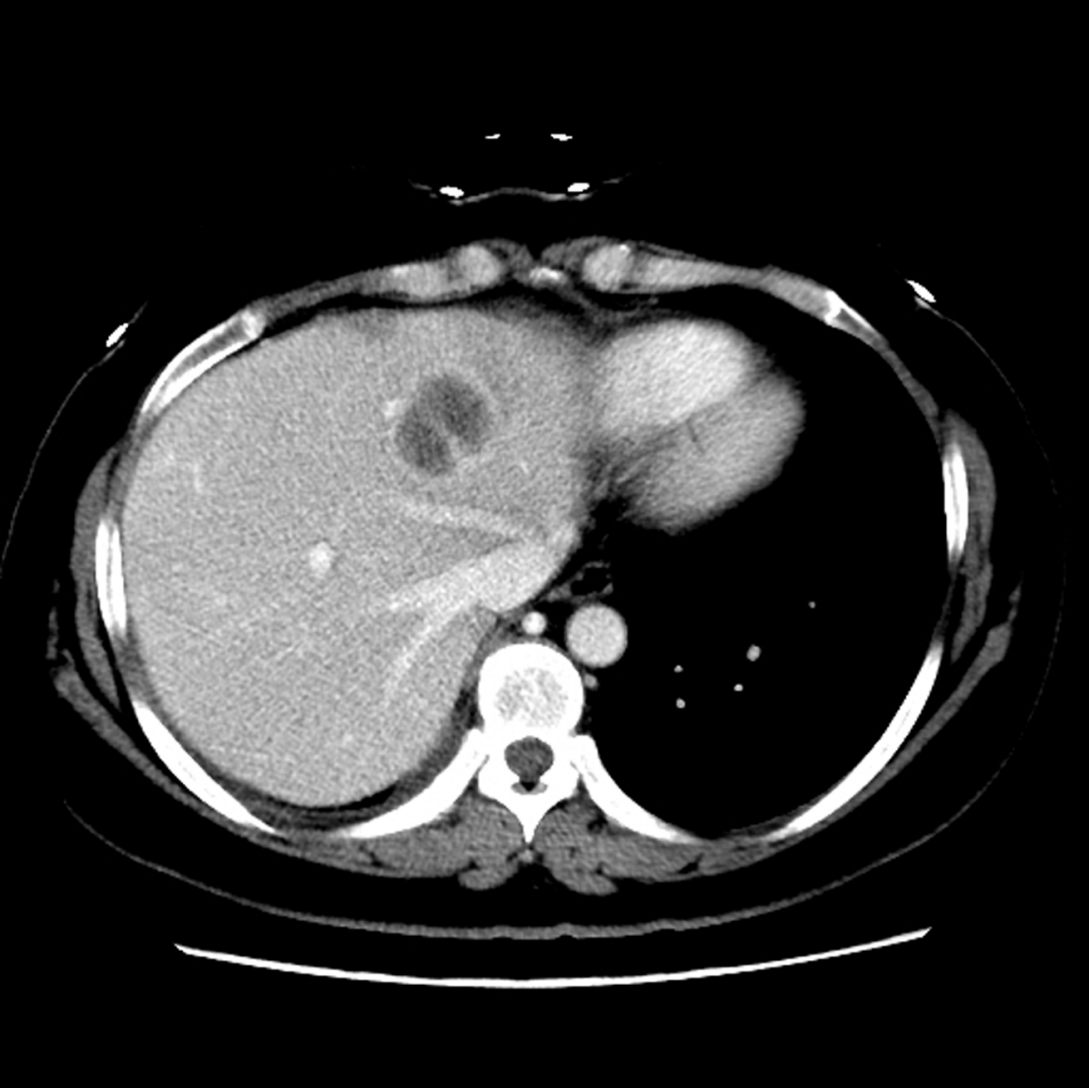
Computed tomography scan images showed new hepatic lesions appeared after 6 months of AZD9291 treatment

**Figure 4:**
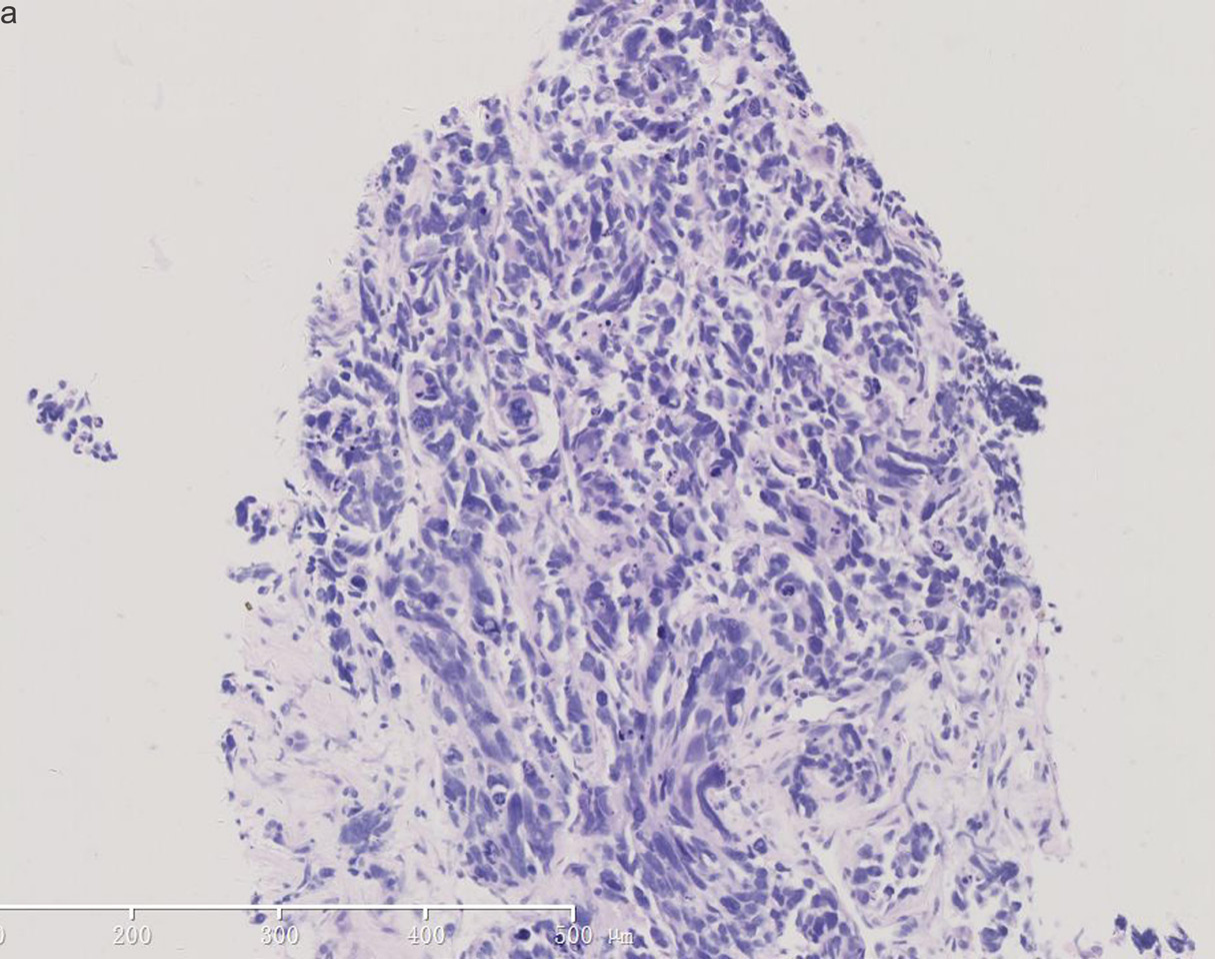
Pathological images of rebiopsy of hepatic mass **a**. HE staining of hepatic mass post-AZD9291 showed small cell carcinoma transformation (X100). **b**. Immunohistochemical staining of hepatic mass post-AZD9291 showed strong synaptophysin staining (+++) (X100).

A 52-year-old non-smoker female was first detected a 2 cm mass in right upper lobe of lung with computed tomography (CT) scan in May, 2014. She then underwent right upper lobectomy with regional lymph node dissection. The pathology diagnosis was adenocarcinoma with multiple metastasized lymph nodes in group 2 (9/9), group 4 (4/4), group 7 (7/7), group 9 (0/1) and group 10 (6/6) (Figure 1). EGFR exon19 deletion was detected by amplification refractory mutation system (ARMS). The patient was diagnosed as adenocarcinoma in right upper lobe, staged T_2_N_2_M_0_ (IIIA). She received adjuvant chemotherapy with gemcitabine plus cisplatin. However, multiple micronodules were found in bilateral lung after finishing two cycles of chemotherapy. Then she started treatment on erlotinib from Sep, 2014 and achieved partial response in one month. Regular CT examination was underwent every two months, and new bilateral lung lesions were found in Aug, 2015, after 11 months treatment of erlotinib. Because of the difficulty of re-biopsy, plasma circulating tumor DNA (ctDNA) was collected for EGFR mutation detection by ARMs. However, neither exon19 deletion nor T790M mutation was detected. The patient was given chemotherapy with pemetrexed plus nedaplatin. But disease progressed after two cycles. Then docetaxol plus bevacizumab was given but disease progressed again. Meanwhile, she had symptoms of cough and shortness of breath. Then she was on AZD9291 in Dec, 2015 after chemotherapy failure. The patient's symptoms improved dramatically in one month and CT scan showed disease improved obviously (Figure 2). She continued on treatment of AZD9291 until multiple hepatic lesions appeared in May, 2016 (Figure 3), while the lesions of lung were still stable. Liver biopsy was performed and histologic analysis showed as small cell lung cancer. Immunohistochemistry staining confirmed as strong positive for synaptophysin (Figure 4). ARMs analysis showed EGFR exon19 deletion, without T790M mutation. Because there were not sufficient tissue left for next generation sequencing assay (NGS) test, peripheral ctDNA was tested and detected mutations of EGFR exon19 deletion, P53 exon6 V203L-pTEN exon4 NC82 and PIK3CA exon10 E545Q. The level of the patient's neuronspecificenolase (NSE) was 113.8 ng/ml, which was 13 ng/ml in Dec, 2015 before treatment of AZD9291. Then the patient was treated on etopside and carboplatin in addition to AZD9291. Her NSE level decreased from 113.8 ng/ml to 28 ng/ml after one cycle of chemotherapy and then to 10 ng/ml after the second cycle. The CT examination after two cycles of chemotherapy showed smaller hepatic lesions but did not reach partial response. Now the patient was on further treatment and followed up.

## DISCUSSION

AZD9291 is one of the third-generation TKIs capable to inhibit mutant EGFR especially after acquired resistance to first-generation TKIs with T790M mutation. Acquired resistance mechanisms to AZD9291 need to be determined. EGFR C797S mutation was reported as one of the mechanisms [4–6]. Tress et al performed droplet digital PCR on serial cell-free plasma DNA specimens collected from fifteen AZD9291 treated subjects positive for T790M mutation before treatment and six cases acquired C797S mutation when resistance occurred [4]. Her2 or MET amplification might also be one of the diversity of resistance mechanisms when tested by NGS [3, 7]. SCLC transformation accounted for acquired resistance to AZD9291 was seldom reported before. As more and more patients were treated with AZD9291, SCLC transformation as resistant mechanism to AZD9291 was also reported. In the article elucidating mechanisms of acquired resistance to AZD9291 by Kim et al in 2015, one patient was found as SCLC transformation [8]. And another two cases of small cell carcinoma transformation were also reported during AZD9291 treatment in 2016 [9]. Here we also reported a case of SCLC transformation after 6 months of AZD9291 treatment.

Small cell carcinoma transformation from NSCLC was reported in first-generation TKIs resistant cases and is accepted as one of acquired resistance mechanisms [1, 10]. Sequist et al reported a rate of 11% of SCLC transformation in resistance to first-generation TKIs treatment [1], although the other reports were not this high. Neuron-specific tumor marker elevation might indicate the happening of transformation in some cases [11, 12]. However, SCLC transformation accounts for acquired resistance to the third-generation TKIs was seldom reported. In our case, the patient's hepatic mass appeared after 6 months of AZD9291 treatment. And her NSE level was normal prior-AZD9291 treatment, and much higher than normal post-AZD9291 treatment when hepatic masses appeared. The NSE level also decreased dramatically after chemotherapy with SCLC regimen.

Our case also has several questions to debate. First, the patient's ctDNA was negative for T790M mutation, but finally she benefitted from treatment of AZD9291. We thought her negative T790M mutation might be a false test result because of the low sensitivity of ARMS in ctDNA detection. Her meanwhile negative exon19 deletion result helped to illustrate it. Oxnard et al explored in 102 patients with T790M-negative plasma using detection of TKI-sensitive EGFR mutation as a control for presence of tumor-derived circulating DNA. Patients with both negative T790M and sensitive mutation could achieve ORR as high as 64% of AZD9291 treatment, which meant a high possibility of false negative. Therefore, testing EGFR sensitive mutation in plasma may help to interpret negative plasma T790M [13]. Secondly, it was questioned whether the hepatic lesions were another primary liver small cell neuronendocrine carcinoma or a histological transformation? We thought it was a transformation because the NSE level was just elevated after AZD9291 treatment and re-biopsy pathology showed small cell carcinoma with EGFR exon19 deletion as well. This case also showed a rapid elevation of serum NSE level accompanied with tumor progression which may indicate SCLC transformation. Detection of persistent elevated NSE level might need attention during EGFR TKIs treatment. Peripheral ctDNA tested by NGS of this case showed mutations of P53, pTEN and PIK3CA. Our NGS detection confirm the previous finding that TP53 mutation accounts for approximately 86% in SCLC [14].

In summary, we deduce that small cell carcinoma transformation is the resistance mechanism to AZD929 for our case. We emphasized the importance of re-biopsy. Both histopathology and molecular test are meaningful, because it may provide more evidence for patient's precise treatment.
